# Ensuring access to affordable, timely vaccines in emergencies

**DOI:** 10.2471/BLT.18.228585

**Published:** 2019-10-28

**Authors:** Elder Kate, Saitta Barbara, Ducomble Tanja, Alia Miriam, Close Ryan, Scheele Suzanne, Erickson Elise, Scourse Rosalind, Kahn Patricia, Elder Greg

**Affiliations:** a Access Campaign, Médecins Sans Frontières, New York, United States of America (USA).; b Médecins Sans Frontières, Geneva, Switzerland.; c Médecins Sans Frontières, Barcelona, Spain.; d Perelman School of Medicine, University of Pennsylvania, Philadelphia, USA.; e Access Campaign, Médecins Sans Frontières, Rue de Lausanne 78, Case Postale 1016, 1211 Geneva 1, Switzerland.; f Médecins Sans Frontières, New York, USA.

Vaccination is an effective intervention to reduce disease, disability, death and health inequities worldwide. Over the last two decades, vaccines have become more accessible in low-income countries; however, significant gaps remain, particularly in humanitarian emergencies, where populations face increased risks of many diseases. In 2013, the World Health Organization (WHO) published *Vaccination in acute humanitarian emergencies: a framework for decision-making*, to provide guidance on which vaccines to prioritize during emergencies.^1^ However, substantial obstacles, especially high prices for new vaccines, hinder implementation of this framework and of critical vaccination activities in emergency settings.

In response to these challenges, global health stakeholders held a series of consultations in 2016 and proposed a WHO-based mechanism, the Humanitarian Mechanism, for the rapid procurement of affordable vaccines during emergencies, to be used by nongovernmental organizations (NGOs), civil society organizations, United Nations (UN) agencies and governments.

Here we present the background of the creation of the mechanism from the perspective of Médecins Sans Frontières (MSF), including a description of our past challenges in accessing affordable pneumococcal conjugate vaccine (PCV; Box 1), a critical vaccine during many emergencies. We then describe how the mechanism has so far facilitated access to more affordable PCV and outline steps that could increase its potential for saving lives.

Box 1 **Timeline of key events leading to the establishment of the Humanitarian Mechanism for affordable, timely access to vaccines in emergencies, 2009–2018**

**June 2009**

Gavi The Vaccine Alliance, launches Advance Market Commitment, with new lowest global PCV price of US$ 3.50 per dose (plus US$ 3.50/dose top-up subsidy) for eligible countries.

**June 2011**

WHO Strategic Advisory Group of Experts constitutes Working Group on Vaccination in Humanitarian Emergencies.

**April 2013**

MSF’s Dear GAVI campaign calls on Gavi, The Vaccine Alliance, to extend its discounted vaccine prices to NGOs and humanitarian actors.

**July 2013**

MSF pays approximately US$ 7.00 per dose for 10-valent PCV to vaccinate refugee children in Yida, South Sudan.

**October 2013**

WHO Strategic Advisory Group of Experts publishes the first edition of *Vaccination in acute humanitarian emergencies: a framework for decision-making*.

**December 2014**

MSF accepts PCV donations, an exception to its donation policy.

**April 2015**

MSF launches A Fair Shot campaign calling on manufacturers to lower PCV prices to US$ 5 per child (for all three doses) for low-income countries and humanitarian organizations.

**May 2016**

MSF pays approximately US$ 68.10 per dose for 13-valent PCV from local pharmacies for a vaccination campaign for refugee children in Greece.

**June and October 2016**

MSF, WHO and other stakeholders^a^ meet to discuss solutions to difficulties in procuring timely supply of affordable vaccines in emergencies.

**September 2016**

GlaxoSmithKline commits to providing its two-dose vial (PCV10) at the lowest global price at the time (US$ 3.05 per dose) to civil society organizations that vaccinate refugees and internally displaced people in emergency settings.

**November 2016**

Pfizer commits to providing its multi-dose (4-dose) vial (PCV13) at the lowest global price at the time (US$ 3.05 per dose) to civil society organizations that vaccinate in emergency settings.

**May 2017**

Humanitarian Mechanism jointly launched by WHO, MSF, UNICEF and Save the Children.

**May 2018**

First monitoring meeting for the Humanitarian Mechanism with representatives from WHO, UNICEF, Save the Children, MSF, Gavi, GlaxoSmithKline and Pfizer.

MSF: Médecins Sans Frontières; NGO: nongovernmental organization; PCV: pneumococcal conjugate vaccine; WHO: World Health Organization; UNICEF: United Nations Children’s Fund; US$: United States dollars.^a^ Other stakeholders included: United Nations High Commissioner for Refugees, UNICEF, International Federation of Red Cross and Red Crescent Societies, Save the Children, Médecins du Monde/Doctors of the World, Centre for Vaccine Ethics and Policy, Gavi, the Vaccine Alliance, International Rescue Committee, International Federation of Pharmaceutical Manufacturers and Associations, Developing Countries Vaccine Manufacturers Network.

## Barriers to affordable PCV

*Streptococcus pneumoniae* is the most common cause of bacterial pneumonia in children and can lead to bacteraemia, meningitis, and other serious conditions, collectively known as invasive pneumococcal disease.^2^ Children caught in humanitarian emergencies are particularly vulnerable to pneumococcal infections when exposed to overcrowding, poor access to health services and malnutrition. PCV, which protects against pneumonia caused by *Streptococcus pneumoniae*, is recommended by WHO for inclusion in immunization programmes for infants up to 23 months of age and children aged 2–5 years who are at high risk of infection.^3^

Before the implementation of the mechanism, neither humanitarian organizations nor countries ineligible for support from Gavi, the Vaccine Alliance, could access the lowest global prices for new vaccines such as PCV. In 2008, MSF stepped up attempts to procure PCV for children in crises; these efforts later included working within the recommendations of WHO’s framework. However, difficulties in sourcing PCV at an affordable price from Pfizer and GlaxoSmithKline, the only two manufacturers of PCVs, undermined our efforts, exemplifying the broader problem of unaffordable vaccines in emergencies, as described in the Global Vaccine Action Plan.^4^ In 2013, MSF added 10-valent PCV (PCV10) and pentavalent vaccines to a mass vaccination campaign package for children in a refugee camp in Yida, South Sudan, two vaccines not in the country’s national immunization schedule at the time. The vaccine products represented 43% (81 040/188 449 United States dollars, US$) of the intervention’s costs, mostly for GlaxoSmithKline’s PCV10 vaccine: its price of approximately US$ 7.00 per dose was nearly four times that of pentavalent vaccine at approximately US$ 1.85 per dose. This cost ultimately led MSF to reduce the planned PCV target population from 6 weeks to 5 years (as recommended by WHO for high-risk populations)^3^ to 6 weeks to 2 years. Similarly, during a PCV vaccination campaign in May 2016 in refugee camps in Greece, MSF found no alternative to buying Pfizer’s PCV13 from local pharmacies, paying up to approximately US$ 68.10 per dose, that is, 20 times its lowest global price.^5^

These examples illustrate how the lack of agreed prices and mechanisms for procuring vaccines in emergencies leads to unpredictable pricing, also causing avoidable delays in launching life-saving vaccination activities, since price negotiations with manufacturers can take months.

Amid ongoing problems and unsuccessful price negotiations, in 2014, MSF made a rare exception to its donation policy^6^ and accepted PCV donations. This exception was made in view of commitments from PCV manufacturers to develop long-term solutions for affordable PCV access. However, no solution was subsequently proposed, and after the donation period concluded, MSF publicly rejected a further PCV13 donation in October 2016.^7^

## The Humanitarian Mechanism

In May 2017, the Humanitarian Mechanism was jointly launched by WHO, the United Nations Children’s Fund (UNICEF), Save the Children and MSF.^8^ The mechanism aims to address the high prices of new vaccines and to facilitate rapid access to vaccines for children in emergencies who missed doses or who live in countries where a given vaccine is not yet in the national immunization calendar, but where there is a high risk of exposure. Under the mechanism’s terms of reference, manufacturers commit to supplying a particular vaccine (currently only PCV10 and PCV13) at a set price. UNICEF, through its supply division, and other organizations with their own supply chain capacity, negotiate contracts bilaterally with manufacturers, specifying ordering and delivery terms as well as protocols. When an emergency need for a vaccine is identified, the implementing organization makes a request to WHO, specifying the context, number of doses needed and other relevant details; within two days WHO verifies the emergency context and appropriateness of the request. Upon verification, the order can be placed and the vaccine supplied.^8^ WHO manages the administrative aspects of the mechanism; once contracts between the implementing organization and manufacturer are in place, the administrative aspects, and the verification process require minimal overhead cost.

## Implementation of the mechanism

In September and November 2016, the two manufacturers announced that they would make their PCV products available through the mechanism at the lowest global price (then US$ 3.05 per dose).^9^^,^^10^ As of April 2019, the mechanism has facilitated access to 764 665 PCV doses for children caught in humanitarian emergencies, 474 402 of which were for 19 MSF vaccination interventions in Central African Republic, Greece, Niger, Nigeria, South Sudan and Syrian Arab Republic. The remaining PVC doses were used by UNICEF, UN Relief and Works Agency, the International Organization for Migration and the Red Crescent. In April 2019, MSF procured 4800 PCV doses for US$ 3.05 each to vaccinate refugee children in Greece, the first use of the mechanism for an emergency in a high-income country.^11^

More broadly, the strategies of MSF in emergencies include vaccinating children up to 59 months old for any vaccine identified by WHO’s framework as a priority in a given context. These strategies include children who did not receive all scheduled doses during their first year of life because the vaccine had not yet been introduced into the national immunization calendar or because of other reasons. Such instances highlight how the framework can be used to expand vaccination age groups in settings where disease burden and outbreak risk are high, and where the mechanism is in place to procure vaccines that are otherwise difficult or impossible to access rapidly and affordably.

## Next steps

Building on these initial successes, we see three critical elements of the mechanism, which if fully implemented, could greatly enhance its impact. First, the mechanism needs to be better known among global health actors, including UN agencies and NGOs, who should be encouraged to use it under appropriate circumstances. Second, while the mechanism’s terms of reference includes use by governments responding to emergencies,^8^ conditions attached to the PCV pledges discussed here (the only pledges to the mechanism so far) exclude governmental use. Manufacturers should allow governments to access the mechanism during emergencies to procure critical vaccines needed to protect their populations. Many middle-income countries already grapple with high vaccine prices for routine immunization programmes and may find the cost of extending vaccination to influxes of displaced people during emergencies prohibitive. While Gavi adopted a fragility, emergencies and refugees policy in June 2017 to allow more flexible use of Gavi-supported vaccine doses in specific contexts, strict criteria exist for how and where this flexibility can be applied, and it can only cover Gavi-eligible countries.^12^

Finally, the types of vaccines pledged to the mechanism by manufacturers should be expanded: the only commitments to date are for pneumococcal conjugate vaccines. Current and future manufacturers should commit other vaccines with affordability and accessibility challenges to the mechanism, so they can be procured rapidly at the lowest global price.

## Conclusion

The mechanism is a significant step forward in delivering life-saving vaccines to populations caught in emergencies and conflict. The mechanism’s mandate is strengthened by multiple organizations recognizing the gap between existing technical guidelines and constraints on their implementation, that is, the lack of rapidly available, affordable vaccines. While the mechanism does not address the broader systemic failures of the vaccine market, it was created to specifically address the failure of the global vaccine market to meet relatively small, urgent vaccine procurement needs efficiently and affordably. Further steps are needed for the mechanism to reach its full potential; however, it already provides a critical platform during humanitarian crises for expanding the number of people who can receive life-saving vaccines.
