# Corrigendum

**DOI:** 10.2471/BLT.20.110220

**Published:** 2020-02-01

In: Elder K, Saitta B, Ducomble T, Alia M, Close R, et al. Ensuring access to affordable, timely vaccines in emergencies. Bull World Health Organ. 2019 Dec 1;97(12):851–53

the author list and affiliations should read as follows:

“Kate Elder,^a^ Barbara Saitta,^a^ Tanja Ducomble,^b^ Miriam Alia,^c^ Ryan Close,^d^ Suzanne Scheele,^a^ Elise Erickson,^e^ Rosalind Scourse,^e^ Patricia Kahn^f^ & Greg Elder^e^

^a^ Access Campaign, Médecins Sans Frontières, New York, United States of America (USA).

^b^ Médecins Sans Frontières, Geneva, Switzerland.

^c^ Médecins Sans Frontières, Barcelona, Spain.

^d^ Perelman School of Medicine, University of Pennsylvania, Philadelphia, USA.

^e^ Access Campaign, Médecins Sans Frontières, Rue de Lausanne 78, Case Postale 1016, 1211 Geneva 1, Switzerland.

^f^ Médecins Sans Frontières, New York, USA.”

